# What’s the best way to leverage 31 million days of ECG data?

**DOI:** 10.1038/s44325-025-00097-z

**Published:** 2025-11-28

**Authors:** L. S. Johnson, S. Z. Diederichsen, E. Svennberg, S. P. Bhavnani, William F. McIntyre, J. S. Healey

**Affiliations:** 1 Department of Clinical Sciences, Lund University, Malmö, Sweden; 2 Population Health Research Institute, McMaster University, Hamilton, ON Canada; 3 Department of Cardiology, Copenhagen University Hospital – Rigshospitalet, Copenhagen, Denmark; 4 Karolinska Institutet, Department of Medicine Huddinge, Karolinska University Hospital, Stockholm, Sweden; 5 Division of Cardiology, Scripps Clinic, San Diego, CA USA; 6 Department of Medicine, McMaster University, Hamilton, ON Canada

**Keywords:** Cardiology, Health care

## Abstract

Smartwatches with pulse-plethysmography sensors capable of detecting irregular rhythms, potentially atrial fibrillation (AF), are increasingly common. Their sensitivity for AF detection in different populations is unknown. If ambulatory ECG monitoring were to be offered to all individuals with notifications this would generate vast amounts of ECG data to analyse. This perspective discusses smartwatch sensitivity for actionable AF, resource utilization to optimize stroke prevention, and suggests alternate ways to manage notifications.

## Smartwatch adoption and large-scale studies

There are an estimated half a billion smartwatches equipped with pulse-plethysmography (PPG) sensors worldwide - around one for every sixteen humans. This number is projected to increase, as it has since the first sensor-equipped smartwatch was introduced in 2013^1^. PPG sensors in the smartwatches and other wearable devices can give notifications to users when an irregular rhythm, which could be atrial fibrillation (AF), is detected.

Three large-scale studies have reported on the notification rates in self-recruited samples of users who had purchased a wearable device: The Apple Heart study (*n* = 419,297, mean age 41 ± 13 years), The Huawei Heart Study (*n* = 187,912, mean age 35 ± 12), and the Fitbit Heart study (*n* = 455,699, median age 47 years, inter-quartile range 35–58 years)^2–4^. The median time that the devices were worn was 4 months in both the Apple Heart Study and the Fitbit Heart Study, while the Huawei Heart Study only specified a minimum wear time of 14 days. During this time, notifications were issued to 0.5% in the Apple Heart Study, 1.0% in the Fitbit Heart Study, and 0.23% in the Huawei Heart Study. In the Apple Heart Study and the Fitbit Heart Study, those who received a notification (0.8% across these two studies) were offered a 7-day ECG-monitoring patch. Only 20.8% and 22.4%, respectively, returned patches for analysis, and among these, AF was detected in approximately a third. In the Huawei study, the workup consisted of a clinical consultation, resting ECG, and 24 h ECG. Given the low mean age among the cohort where AF was detected (59 years in the Apple Watch Study, and 56 years in the Huawei Heart Study) many of these patients with newly detected AF would have such a low risk of stroke that they would not benefit from oral anticoagulation^5^. While there is a potential that early identification of AF with wearables, and subsequent interventions could potentially lead to other benefits, such as risk factor management or rhythm control to prevent AF progression or heart failure^6^, there is as yet no prospective data that shows an effect of interventions in patients with wearable detected AF.

## Sensitivity and limitations of PPG monitoring

The low notification rates in the studies are to be expected due to the younger age of the study populations, as exemplified by the much higher notification rate in the subgroup of ≥65-year-olds of 3.1% in the Apple Heart Study^3^, and 3.6% in the Fitbit study, but it could also be due at least in part to the monitoring strategies of the wearables. Wearables display heart rates in real time, but they do not monitor the heart rate continuously. First, because intermittent sampling preserves computing power and energy, and second, because sampling is generally disabled when signal quality is deemed below threshold, i.e., during movement as defined by accelerometery, poor fit of the wristband, or during device-estimated non-wear. As an example, the Apple Watch algorithm described in the Apple Heart Study analyses one minute of PPG every 2 hours, and increases the number of samples to one per 15 minutes in case an irregular rhythm is detected. When five out of six samples are above the irregularity threshold, an irregular rhythm notification is triggered^3^. Details of subsequent versions of the algorithm have not been published, nor has the minimum detectable duration of AF, or the percent of time when samples are not collected due to motion or other issues with sample collection. If one assumes that the wearer is in AF the entire time that would imply a minimum duration of 1 hour and 15 minutes, but since the monitoring is intermittent, different repeated patterns of shorter episodes, and perhaps also an interspersion of other irregular rhythms such as sinus arrhythmia or premature ventricular or supraventricular beats, could theoretically also trigger a notification. None of the studies that report on AF detection using wearables have reported the sensitivity of the PPG devices compared to continuous monitoring data in the full study population, but given the intermittent monitoring strategy, and the low notification rates, one can assume that there is a substantial amount of AF cases that remains undetected, especially those with episodes that last less than a few hours. This is supported by the fact that only 67.6% of patients with AF detected during ECG monitoring in the Fitbit Heart Study had notifications at the time of AF, possibly an effect of movement during the time of AF hampering the PPG monitoring, and short episode durations. Low notification rates are therefore not only explained by the relatively low age of the study populations, but also by an under-detection of AF.

## Downstream effects of irregular rhythm notifications

If we assume that all who receive an irregular rhythm notification should be monitored with a 7-day ECG patch, as in the Apple Heart study and the Fitbit Heart study, the average notification rates across these studies would result in 31 million days of ECG (notification rate 0.8% in 560 million users, with seven days of monitoring each) that need to be done worldwide, and this would result in confirmation of 3.5 million cases of AF. This is based on the follow-up times in the study, which are less than a year, and would increase over time. The need for ECG monitoring would increase further if PPG were improved to detect also shorter AF episodes or episodes during physical movement, as noted above. A method for confirming AF as in The Huawei Heart would lead to a large number of clinical consultations. In contrast, among Swedish 75–76 year-olds, the intermittent monitoring strategy in the STROKESTOP trial (twice daily for 14 days, using a handheld ECG device), resulted in the detection of AF in 3.0% of the study population, and this was shown to reduce the primary composite endpoint of stroke, systemic embolism, bleeding, and death using intention-to-screen analyses^7,8^.

ECG monitoring is a finite resource, with a capacity determined by the cost of human analysis, and a global shortage of trained healthcare staff^9^. The ECG monitoring that is performed to confirm or rule out AF in individuals who have received an irregular rhythm notification is done within the framework of the regular health care system, and the costs for this monitoring are thus borne by the regular budget in any single-payer system, or by the insurer in a private insurance system. To know whether the monitoring driven by wearable use is rational and meets key population health performance metrics, a health system has to consider the diagnostic yield on a workflow of the smartwatch-driven prescription of 7-day ECG patches in the context of the detection rates when monitoring is done for clinical reasons. When applying continuous ECG monitoring to unselected patient populations, the detection rate for AF over 7-days of monitoring is 9-10% in both sexes, and less than half of the paroxysmal AF cases that occur in clinical populations have been detected after 24–48 h of monitoring^10,11^. In post stroke patients the AF prevalence is higher, between 15 and 24%^12,13^. Consensus documents recommend at least 72 h of ECG monitoring in post-stroke patients, but, due to limitations in access to care and costs, shorter durations are common, despite this being a patient population with high stroke risk that likely benefits from AF detection. In this context, it is questionable whether resource allocation to low-risk patients with irregular rhythm notifications from wearables is justified. The problem could be mitigated by using AI-only analysis of ECG recordings, with human intervention only in case of detection of a clinically important arrhythmia. This has been shown to be feasible in the recent DRAI MARTINI study, which tested AI-only analysis for direct-to-physician reporting of ambulatory ECG recordings, and found a 14-times lower risk of false negatives for DeepRhythmAI compared to ECG technicians, with an acceptable false positive rate of 12 per 1000 recording days for DeepRhythmAI compared to 5 per 1000 recording days for the technicians. Assuming the same rate of false positives in a population with irregular rhythm notifications and 7-day monitoring to detect or rule out AF this would imply a false positive result in 8% with DeepRhythmAI, compared to 3% for technicians^14^. An approach where patients with irregular rhythm notifications and high risk of stroke or heart failure are offered long-term monitoring, but with AI-only analysis of the ECG recording using a validated state-of-the art model, could thus be a cost-effective and scalable solution that does not compromise patient safety. Studies of integration of AI analysis in clinical workflow are needed. A potential drawback is that state-of-the-art AI models use considerable amounts of computer processing power and electricity. Since many wearables are also capable of recording a single-lead ECG, another use of AI that could increase real-world performance would be to prompt the user to record an ECG on the watch, analyse it using AI, and only forward to the healthcare system ECGs in which the AI detects arrhythmias. At the very least, robust evidence that meets regulatory requirements is needed to confirm that wearables PPG technologies are safe and effective as diagnostic devices, not only as a bridge to continuous ECG monitoring as it is presently used. Future studies are needed to assess smartwatch sensitivity for AF detection, so that important clinical questions can be answered, for example whether a smartwatch can be used to rule out AF at different levels of burden and duration. Currently available evidence indicates that they should not be. While several commercially available monitoring devices have both PPG and ECG-based sensors, few head-to-head comparative evaluations on performance and detection rates of PPG vs ECG have been performed. While PPG based-devices may have a greater pooled sensitivity (97% vs. 83%) and specificity (97% vs. 83%) compared to ECG-based devices^15^, marked heterogeneity exists from factors including the quality of the signal analyzed to the rate of inconclusive ECG tracings that can reduce the sensitivity and specificity of any given smartwatch for ECG-based detection of AF^16^.

## Conclusions

In conclusion, we see several problems related to the increasing use of wearables. Their sensitivity for the detection of AF in patients in whom such a diagnosis would be considered actionable in terms of stroke prevention is unknown, and there is therefore a risk that wearables may give a false sense of security; actionable AF cases may not be detected. As there is a finite number of Holter and patch-based monitoring devices in any given clinic or healthcare facility, there is a need for studies that address whether smartwatch-driven monitoring reduces the availability of conventional patch-based devices for patients who would benefit more. Several studies have shown that short-duration AF is associated with increased stroke risk in patients with high CHA_2_DS_2_-VASc scores^5,17,18^, and in post-stroke patients; these cases may be missed by the intermittent monitoring strategies of the algorithms. At the same time, the wearables issue a vast number of irregular rhythm notifications that lead to a need for ECG monitoring to confirm or rule out AF. If we consider that there is an upper limit to the amount of rhythm analysis that can be done, the millions of days of ECG monitoring that wearables generate could probably be better spent on extending ECG monitoring in patients with a clinical indication.

## Data Availability

No datasets were generated or analysed during the current study.

## References

[1] https://www.demandsage.com/smartwatch-statistics/, Access date 16 May 2025

[2] Lubitz, S. A. et al. Detection of Atrial Fibrillation in a Large Population Using Wearable Devices: The Fitbit Heart Study. *Circulation* **146**, 1415–1424 (2022). 36148649 10.1161/CIRCULATIONAHA.122.060291 PMC9640290

[3] Perez, M. V. et al. Large-Scale Assessment of a Smartwatch to Identify Atrial Fibrillation. *N. Engl. J. Med.* **381**, 1909–1917 (2019). 31722151 10.1056/NEJMoa1901183 PMC8112605

[4] Guo, Y. et al. Mobile Photoplethysmographic Technology to Detect Atrial Fibrillation. *J. Am. Coll. Cardiol.* **74**, 2365–2375 (2019). 31487545 10.1016/j.jacc.2019.08.019

[5] Lopes, R. D. et al. Apixaban versus Aspirin According to CHA2DS2-VASc Score in Subclinical Atrial Fibrillation: Insights from ARTESiA. *J. Am. Coll. Cardiol.* **84**, 354–364 (2024). 39019530 10.1016/j.jacc.2024.05.002

[6] Van Gelder, I. C. et al. 2024 ESC Guidelines for the management of atrial fibrillation developed in collaboration with the European Association for Cardio-Thoracic Surgery (EACTS). *Eur. Heart J.* **45**, 3314–3414 (2024). 39210723 10.1093/eurheartj/ehae176

[7] Svennberg, E. et al. Clinical outcomes in systematic screening for atrial fibrillation (STROKESTOP): a multicentre, parallel group, unmasked, randomised controlled trial. *Lancet* **398**, 1498–1506 (2021). 34469764 10.1016/S0140-6736(21)01637-8

[8] Svennberg, E. et al. Mass Screening for Untreated Atrial Fibrillation: The STROKESTOP Study. *Circulation* **131**, 2176–2184 (2015). 25910800 10.1161/CIRCULATIONAHA.114.014343

[9] Mathieu, B. et al. The global health workforce stock and distribution in 2020 and 2030: a threat to equity and ‘universal’ health coverage?. *BMJ Glob. Health* **7**, e009316 (2022). 10.1136/bmjgh-2022-009316 PMC9237893 35760437

[10] Dziubinski, M. et al. Diagnostic yield is dependent on monitoring duration. Insights from a full-disclosure mobile cardiac telemetry system. *Kardiol. Pol.* **80**, 49–55 (2022). 34913475 10.33963/KP.a2021.0182

[11] Steinhubl, S. R. et al. Effect of a Home-Based Wearable Continuous ECG Monitoring Patch on Detection of Undiagnosed Atrial Fibrillation: The mSToPS Randomized Clinical Trial. *JAMA* **320**, 146–155 (2018). 29998336 10.1001/jama.2018.8102 PMC6583518

[12] Jabaudon, D., Sztajzel, J., Sievert, K., Landis, T. & Sztajzel, R. Usefulness of ambulatory 7-day ECG monitoring for the detection of atrial fibrillation and flutter after acute stroke and transient ischemic attack. *Stroke* **35**, 1647–1651 (2004). 15155965 10.1161/01.STR.0000131269.69502.d9

[13] Sposato, L. A. et al. Diagnosis of atrial fibrillation after stroke and transient ischaemic attack: a systematic review and meta-analysis. *Lancet Neurol.* **14**, 377–387 (2015). 25748102 10.1016/S1474-4422(15)70027-X

[14] Johnson, L. S. et al. Artificial intelligence for direct-to-physician reporting of ambulatory electrocardiography. *Nat. Med.* **31**, 925–931 (2025). 10.1038/s41591-025-03516-x PMC11922735 39930139

[15] Luo, D. Y., Zhang, Z. W., Sibomana, O. & Izere, S. Comparison of diagnostic accuracy of electrocardiogram-based versus photoplethysmography-based smartwatches for atrial fibrillation detection: A Systematic Review and Meta-Analysis. *Ann. Med Surg. ((Lond.))* **87**, 2307–2323 (2025). 40212135 10.1097/MS9.0000000000003155 PMC11981249

[16] Mannhart, D. et al. Clinical Validation of 5 Direct-to-Consumer Wearable Smart Devices to Detect Atrial Fibrillation. *JACC: Clin. Electrophysiol.* **9**, 232–242 (2023). 36858690 10.1016/j.jacep.2022.09.011

[17] McIntyre, W. F. et al. Estimated incidence of previously undetected atrial fibrillation on a 14-day continuous electrocardiographic monitor and associated risk of stroke. *Europace*. **24**, 1058–1064 (2022). 10.1093/europace/euab324 35061877

[18] Kaplan, R. M. et al. Stroke Risk as a Function of Atrial Fibrillation Duration and CHA2DS2-VASc Score. *Circulation* **140**, 1639–1646 (2019). 31564126 10.1161/CIRCULATIONAHA.119.041303

